# Analysis of column reactor results with organic decay by native organic microbiota and varying permeability

**DOI:** 10.1038/s41598-021-84530-0

**Published:** 2021-03-03

**Authors:** Fernanda Costa da Silva Maciel, Sandro Lemos Machado, Antonio Fernando de Souza Queiroz, Fernando Antonio Leite Vieira Lima

**Affiliations:** 1 Department of Materials Science and Technology, Federal University of Bahia, Salvador, 40.210-630 Brazil; 2 Geo-Science Institute, Federal University of Bahia, Salvador, 40.210-630 Brazil

**Keywords:** Environmental biotechnology, Biogeochemistry, Environmental sciences, Solid Earth sciences, Civil engineering

## Abstract

Field bio-remediation techniques (FBRT) can be a low cost method to avoid the removal of top layers of soil which are rich in organic matter and bio diversity. The use of native microorganisms in FBRT is preferable because non-indigenous species can transfer their genetic material to the environment with negative impacts on the local ecological equilibrium. Petroleum Produced Water (PPW) is an important pollutant source in onshore production areas. However, due to high sodium concentrations in PPW and the occurrence of organic matter in dissolved and dispersed forms, obtaining pollutant transport parameters may be a difficult task. Results of column tests performed using a natural soil permeated by PPW are presented. All the samples presented a permeability decrease over time and the total hydrocarbon petroleum (TPH) breakthrough curves presented evidence of biological decay. Soil samples underwent biological characterization after tests (Metagenomic analyses and cultural media tests). Curves were modelled in an incremental way using a non-constant decay rate to better simulate the growing process of the microorganisms and consider the occurrence of varying velocity/permeability. Biological characterization results indicate the native organisms that are potentially more able to degrade PPW, including four bacteria (*Bacillus* and *Lysinibacillus* genus) and two fungi species (*Malassezia* and *Talaromyces* genus) that have not previously been mentioned in the consulted literature. The obtained results contribute to the development of more sustainable FBRTs focusing on native microorganisms, already adapted to the local environmental conditions.

## Introduction

Pollutant transport parameters are very important when evaluating the impact of accidental leakages in onshore facility areas or selecting appropriated remediation technologies. Knowledge of the involved phenomena and acquisition of the necessary variables for problem modeling enable a better understanding of the transport and fate of the released pollutant components. Equation 1 can be used to describe solute transport in soils in transient fluid flow conditions^1^:

1 $$\begin{aligned} \dfrac{\partial (R_d C)}{\partial t} = \nabla \cdot \left( \theta D_h \nabla C -v_s C \right) -\lambda \theta C \end{aligned}$$

where $\mathrm{C}$ is the solute concentration ($\mathrm{M}\cdot \mathrm{L}^{-3}$), $\uptheta$ is the volumetric water content ($\mathrm{L}^3\cdot \mathrm{L}^{-3}$), $\mathrm{D}_{\mathrm{h}}$ is the hydrodynamic dispersion coefficient ($\mathrm{L}^2\cdot \mathrm{T}^{-1}$), $\mathrm{v}_{\mathrm{s}}$ is the average (macroscopic) fluid velocity ($\mathrm{L}\cdot \mathrm{T}^{-1}$), and $\mathrm{t}$ is time (T). $\uplambda$ is the rate constant for first-order decay ($\mathrm{T}^{-1}$) and $\mathrm{R}_{\mathrm{d}}$ is the retardation factor. $\mathrm{D}_{\mathrm{h}}$ (refer to Eq. 2) includes the effects of both mechanical dispersion ($\mathrm{D}_{\mathrm{m}}$) and molecular diffusion ($\mathrm{D}^*$) in soil.

2 $$\begin{aligned} D_h=D^*+D_m \end{aligned}$$

Solute dispersion is a consequence of the heterogeneity of the soil on a microscopic scale and $\mathrm{D}_{\mathrm{m}}$ is normally calculated as a linear function of $\mathrm{v}_{\mathrm{s}}\,(\mathrm{L}\cdot \mathrm{T}^{-1})$ (refer to Equation 3).

3 $$\begin{aligned} D_{m}=\alpha _L v_s \end{aligned}$$

Pollutant transport parameters in soil are normally evaluated using column test apparatus^2–4^. In some cases, however, a decrease in the soil permeability coefficient (k) has been observed despite keeping boundary conditions constant and the changes in the k values are normally attributed to physical, chemical, and biological phenomena such as filtration, bio-clogging, and clay dispersion (CD)^5^. Clay dispersion is an important theme in soil sediment, petroleum engineering and agronomy^6–9^ and occurs when soil is submitted to sodium-water flow, because sodium can deteriorate the soil structure^7^, expanding and disaggregating the clay particles and partially filling or blocking soil pores^10^. CD is normally pointed as the main mechanism controlling k reduction when soil is permeated by PPW, a sodium residue of petroleum production activities^11^. Furthermore, soils permeated by PPW normally present high ${\mathrm{D}}_{\mathrm{h}}$ values^11, 12^ compared with those for inorganic dissolved species^13, 14^. The presence of dissolved and dispersed organic compounds in PPW tends to increase $\mathrm{D}_{\mathrm{h}}$ values and partially justifies the very high observed $\upalpha _{\mathrm{L}}$ values^12^.

The adoption of FBRT could be very attractive and low-cost and avoid the removal of most superficial soil which is usually rich in organic matter and microorganism diversity. The occurrence of highly permeable substrates, which facilitates the injection and transit of the desired microorganisms in subsurface is desirable. For superficial soil, phytoremediation technology can be used as long-term method to eliminate heavy metals^15^. Although the use of FBRT has drawn the attention of many researchers in recent years, the efficiency of these techniques is dependent on several environmental variables (poor capabilities of microbial communities in the field, lesser bioavailability of pollutants, growth conditions, etc.) and their use is not always suitable^16^. Furthermore, because the range of contaminants on which bioremediation is effective is limited, the time scales are relatively long, and the residual contaminant levels may not be acceptable^17, 18^. The use of native microorganisms is preferable to the use of non-indigenous microorganisms because they can reproduce, spread to new locations, and even transfer genetic material to microorganisms that naturally exist in the environment.

The study of microbial abundance and distribution in natural environments traditionally makes use of culture-dependent methods, which are based on metabolic and physiological characteristics. These characteristics include isolation and cultivation using solid and/or liquid media. However, with the rapid progress in molecular biology, polymerase chain reaction (PCR)-based approaches have emerged to study specific microorganisms or groups of microorganisms^17^.

In this paper the results of column tests performed on large samples of compacted soil are analysed to evaluate the occurrence of organic decay and their hydraulic behaviour during the percolation of PPW. The proposed experimental program consisted of performing column tests, measuring the permeability coefficient and analysing the effluent properties to plot the breakthrough curves. The chemical analyses involved the determination of parameters such as the TPH and cations concentrations, pH and electrical conductivity (EC). Furthermore, in an interdisciplinary effort, after breakthrough curve stabilization, soil samples were collected and submitted to metagenomic analyses and traditional cultural media tests to better characterize the organic processes and organisms that are involved in the degradation of PPW components.

## Materials and methods

### Soil

Table 1 presents the studied sediment (*Várzea soil, VS*) geotechnical characterization. VS is a fluvial sediment located on the banks of an intermittent river that crosses an onshore petroleum production area in northeast Brazil. Two sampling campaigns were performed in the field. The first campaign focused on the occurrence of clay dispersion during the tests, whereas the second campaign focused on the microorganisms that control the observed organic decay. As can be observed, the soil samples presented similar characterization results. The samples constituted a clayey sand classified as $\mathrm{SC}$ by USCS (Unified Soil Classification System). Complementary characterization tests, such as X-ray diffraction (XRD) (material passing on $\#200$), X-ray fluorescence (XRF), and total volatile solids (TVS), were also performed. The main soil constituent elements (apart from oxygen) were $\mathrm{Si}=27.8\%$, $\mathrm{Al}=7.2\%$, $\mathrm{Ca}=3.32\%$, $\mathrm{K}=3.1\%$, and $\mathrm{Cl}=3.7\%$ whereas quartz (20.4%), kaolinite (14.2%), microline (13.2%), and calcite (9.4%) were the main soil minerals (amorphous phase of 35.2%). An average total volatile solids value of $\mathrm{TVS}=3.9\%$ was obtained.

**Table 1:** Soil geotechnical characterization.

Soil sample	Particle size distribution (%)				Altterberg limits (%)			$\mathrm{A}_{\mathrm{c}}$	$\uprho _{\mathrm{s}}$ ($\mathrm{g} \cdot \mathrm{cm}^{-3}$)	Classification	Compaction properties normal proctor	
	Gravel	Sand	Silt	Clay	$\mathrm{W}_{\mathrm{L}}$	$\mathrm{W}_{\mathrm{P}}$	PI			USCS	$\mathrm{w}_{\mathrm{ot}}$ (%)	$\uprho _{\mathrm{dmax}}$ ($\mathrm{g} \cdot \mathrm{cm}^{-3}$)
Várzea soil$^1$	1	50	14	35	48	22	26	0.74	2.73	SC	15.6	1.67
Várzea soil$^2$	1	55	12	32	46	25	21	0.66	2.73	SC	18.1	1.66

Obs: $\mathrm{A}_{\mathrm{c}}$-soil activity; $^{1,2}$: samples from first and second sampling campaigns.

### Petroleum produced water

Samples collected in the first field campaign were permeated by PPW from a nearby well. Samples from the second campaign were permeated by a synthetic PPW generated using the oil phase from the previous PPW. Table 2 presents the average PPW properties.

**Table 2:** PPW main properties.

Column test	TPH ($\upmu \mathrm{g/L}$)	EC $(\upmu \mathrm{S/m})$	pH	K (mg/L)	Mg (mg/L)	Ca (mg/L)	Na (mg/L)	Salinity (mg/L)	$\mathrm{T}_{\mathrm{\mathrm s}}\,(20\,^{\mathrm{o}}\mathrm{C})$ (mN/m)	$\upmu \,(20\,^{\mathrm{o}}\mathrm{C})$ cP
PPW $^1$	2860	2540	7.0	15	45	15	277	1320	–	1.36
PPW $^2$	1117	2380	6.12	–	–	–	390	1350	65.1	1.35

EC = electrical conductivity; $\mathrm{T}_{\mathrm{s}}$ = superficial tension; $\upmu$ = viscosity; cP = centipoise.

$^{1,2}$; samples from first and second sampling campaigns.

### Column tests

**Figure 1:**
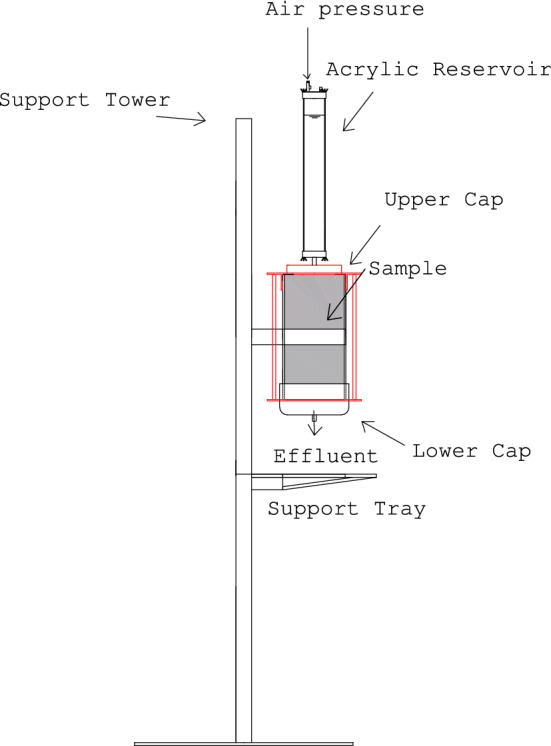
Sketch of the column test device.

Figure 1 presents the experimental apparatus. The columns are made up of a reservoir, tower and percolation column. The compacted specimens had approximate dimensions of $\upphi = 20\,\mathrm{cm}$ (diameter) and $\mathrm{L} = 40\,\mathrm{cm}$ (height). Before the tests, samples were saturated with water and the acrylic top reservoir was greased using PPW oil to avoid absorption of organic compounds by the tube wall. At top and bottom caps of the columns, filters composed of a mixture of coarse sand and gravel were installed. Soil samples were compacted directly inside the stainless steel body of the columns which had their inner surface previously coated with epoxy and covered with a thin layer of sand ensuring mineralogical similarity with the soil and preventing the occurrence of preferential flow paths.

Table 3 presents the characteristics of the samples after compaction. $\mathrm{w}$ is the gravimetric water content. All the column tests were initiated following the falling head procedure (use of atmospheric pressure on the top of the acrylic reservoir). However, owing to the observed decrease in the values of $\mathrm{k}$, higher energy gradients were employed when values of permeability coefficient were lower than $1 \times 10^{-9}\,\mathrm{m} \cdot \mathrm{s}^{-1}$. In this case, the air pressure applied to the top of the acrylic reservoir (refer to Fig. 1) was increased. Table 4 presents additional information about the performed tests. During the tests, effluent samples were collected continuously at the base of the columns using 250 $\mathrm{cm}^3$ glass vials.

**Table 3:** Soil samples compaction conditions and phase relationships.

Soil specimen	Compaction		$\mathrm{w}-\mathrm{w}_{\mathrm{ot}}$	$\uprho _{\mathrm{d}}/\uprho _{\mathrm{dmax}}$	$\uptheta (\%)$	Sr (%)	n	Void volume ($\mathrm{cm}^3$)
	w (%)	$\uprho _{\mathrm{d}}\,(\mathrm{g}/\mathrm{cm}^3)$						
CP 1$^1$	14.8	1.61	− 0.77	0.98	23.8	58.2	0.41	4736
CP 2$^1$	15.1	1.61	− 0.51	0.98	24.3	59.2	0.41	4754
CP 3$^1$	14.0	1.61	− 1.55	0.98	22.6	55.1	0.41	4749
CP 1$^2$	19.1	1.66	− 1.02	1.00	31.45	80.7	0.39	5443
CP 2$^2$	18.4	1.67	− 0.27	1.01	30.23	77.5	0.39	5390
CP 3$^2$	19.1	1.66	− 0.96	1.00	31.36	80.4	0.39	5439

$^{1,2}$: samples from first and second sampling campaigns.

**Table 4:** Main hydraulic characteristics of the performed column tests.

Soil sample	Average hydraulic head (m)	Average gradient	Average discharge of flow ($\mathrm{m} \cdot \mathrm{s}^{-1}$)	Time (days)
CP 1$^1$	0.70	1.75	2.8E−08	224.84
CP 2$^1$	0.72	1.80	2.4E−08	224.98
CP 3$^1$	0.78	1.94	1.8E−08	224.88
CP 1$^2$	22.7	56.8	1.1E−08	194.00
CP 2$^2$	42.8	106	1.2E−08	166.00
CP 3$^2$	23.0	57.6	1.3E−08	230.00

$^{1,2}$Samples from first and second sampling campaigns.

Effluent samples were kept at 5 $^{\circ }\mathrm{C}$ until chemical characterization. A minimal volume of $500\,\mathrm{cm}^3$ (or $0.092\,\hbox {Vp}$, pore volume) was required for analytical tests (use of two consecutive samples). TPH concentrations were determined following the EPA 8015B method with the quantification of the n-alkanes (n-C12 to n-C40), isoprenoids (phytane and pristane), resolved compounds and unresolved complex mix (UCM).

Breakthrough curves ($\mathrm{C}/\mathrm{C}_0 \,\times \,\mathrm{V}_{\mathrm{p}}$) were fitted using Eq. (4)^1^ which is the solution of Eq. (1) considering organic decay, unidirectional flow and the following boundary conditions: $\mathrm{C}(\mathrm{z},0)=0\,\mathrm{p}/\,\mathrm{z}>0$, $\mathrm{C}(0,\mathrm{t})=\mathrm{C}_0\,\mathrm{p}/\,\mathrm{t}\ge 0$, and $\mathrm{C}(\infty ,\mathrm{t})=0\,\mathrm{p}/\,\mathrm{t}\ge 0$. The equation was employed in an incremental way owing to the variations in the $\uplambda$ and $\mathrm{v}_{\mathrm{s}}$ values during the tests.

4 $$\begin{aligned} \dfrac{dC}{C_0} = \dfrac{1}{2} \left[ \begin{array}{c} \dfrac{-2}{\sqrt{\pi }}\, e^{x_1}\, e^{{-x_2}^2}\, dx_2+erfc(x_2)\, e^{x_1}\, dx_1...\\ -\dfrac{2}{\sqrt{\pi }}\, e^{{-x_4}^2}\, e^{x_3}\, dx_4 +erfc(x_4)\, e^{x_3}\, dx_3 \end{array} \right] \end{aligned}$$

where

5 $$\begin{aligned} x_1= & {} \dfrac{(v_s -u)\cdot z}{2\cdot D_h} \end{aligned}$$

6 $$\begin{aligned} x_2= & {} \dfrac{R_d \cdot z -u \cdot t}{2 \cdot \sqrt{D_h \cdot R_d \cdot t}} \end{aligned}$$

7 $$\begin{aligned} x_3= & {} \dfrac{(v_s +u)\cdot z}{2\cdot D_h} \end{aligned}$$

8 $$\begin{aligned} x_4= & {} \dfrac{R_d \cdot z +u \cdot t}{2 \cdot \sqrt{D_h \cdot R_d \cdot t}} \end{aligned}$$

9 $$\begin{aligned} u= & {} v_s \sqrt{1+\dfrac{4 \cdot \lambda \cdot D_h}{{v_s}^2}} \end{aligned}$$

In order to better simulate the growing process of the microorganisms Eq. (10)^11^ was used to model the $\uplambda$ behavior during the tests.

10 $$\begin{aligned} \lambda =\dfrac{\lambda _{max}}{1+e^{-\beta (t-t_1)}} \end{aligned}$$

where $\uplambda _{\mathrm{max}}$ corresponds to the maximum decay rate, obtained after microorganism population stabilization, $\upbeta$ is the population growth rate, and $\mathrm{t}_1$ corresponds to the time for which $\uplambda =\uplambda _{\mathrm{max}}/2$.

The following procedures were adopted to estimate the fitting variables guess values: The $\upalpha _{\mathrm{L}}$ values were estimated using Eq. (11)^19^.

11 $$\begin{aligned} \alpha _L= \dfrac{{v_s}^2}{4 \pi L \omega ^2} \end{aligned}$$

where $\mathrm{L}$ is the sample length and $\upomega$ is the slope of the breakthrough curve between $\mathrm{C}/\mathrm{C}_0=0.25$ and $\mathrm{C}/\mathrm{C}_0=0.5$ ($\upomega =\Delta (\mathrm{C}/\mathrm{C}_0)/\Delta \mathrm{t}$). The diffusion coefficient was estimated considering the PPW number of equivalent carbons, $\mathrm{N}_{\mathrm{ec}}$^20^. A value of $\mathrm{D}^*=5.1 \times 10^{-6}\,\mathrm{cm}^2/\mathrm{s}$ was calculated considering $\mathrm{N}_{\mathrm{ec}}=10$, value obtained from the fluid characterization results and soil tortuosity. Due to the high $\upalpha _{\mathrm{L}}$ values, however, the diffusion coefficient had a minor effect on the fitted results. $\mathrm{R}_{\mathrm{d}}$ was initially set as $\mathrm{R}_{\mathrm{d}}=1$ (minimum value considered).

A reasonable estimation for $\mathrm{t}_1$ was the time necessary to reach a peak in the breakthrough curve, and the value of $\mathrm \uplambda _{\mathrm{max}}$ directly influences the $\mathrm{C}/\mathrm{C}_0$ values for higher values of $\mathrm{V}_{\mathrm{p}}$. Appropriate $\upbeta$ guess values were obtained according to the expression $\upbeta =(\mathrm{t}_2-\mathrm{t}_1)^{-1}$, where $\mathrm{t}_2$ corresponds to the time for which $\mathrm{C}/\mathrm{C}_0$ becomes approximately constant.

The breakthrough curves were fitted according to the minimum squares method in an interactive process (use of the Libreoffice (6.4.2) solver), applying the restriction $\mathrm{R}_{\mathrm{d}}\ge 1$. A good choice of the initial values of the fitting variables is important because the fitting process may converge to high $\mathrm{R}^2$ despite using physically unrealistic combinations of the pollutant transport parameters.

### Biological characterization

Biological characterization of the soil samples in the second campaign was performed. After breakthrough curve stabilization, the columns were opened and the soil samples (200 $\mathrm{g}$) were collected at different depths to study the microbial abundance and diversity. Samples of *in natura* soil (reference values) were also submitted to the same procedures. Samples were quartered, and part of the collected material was frozen and stored in plastic vessels ($-\,20\,^{\circ }\mathrm{C}$) for genetic analysis. The remaining material was kept refrigerated in aluminum recipients for a maximum 24 h for culture and isolation tests.

The serial dilution and cultivation procedures were performed incubating the plates containing aliquots of the dilutions at 30 $^{\circ }\mathrm{C}$ for 24 h and 7 days to count the colony forming units (CFU) of bacteria and fungi, respectively^21^. Eighty-two different types of microorganisms were visually identified, isolated, and then submitted to oxidation tests (two stages)^22^. Tests used PPW as the carbon source, Bushnell-Haas medium (Difco), microbial suspension and redox indicator 2,6-dichlorophenolindophenol (DCPIP). Only the organisms with more than 50% efficiency in the first phase were submitted to the second phase of oxidation. Forty-one microorganisms were identified with the potential to degrade the PPW (100% of efficiency in the second phase according to the redox indicator) and submitted to DNA sequencing procedures for gender identification. Samples of PPW were submitted to the cultivation procedure. Due to the high salinity and low nutritional content of the PPW, however, it was not necessary to perform filtration or dilution procedures to obtain quantifiable CFU values of fungi and bacteria.

In the genetic identification of the microorganisms that fit the second oxidation phase requirements approximately 50 $\mathrm{mg}$ of cells were used in the DNA extraction method (DNeasy UltraClean microbial kit), which afford the isolation of genomic DNA from the microbial cultures. Full-spectrum NanoDrop 2000 was employed to assess the purity of the extracted DNA. The DNA amplification was performed by PCR in a Veriti thermal cycler by a reaction that employs 20 $\mathrm{ng}$ of purified genomic DNA. The PCR products were analysed by electrophoresis and subsequently purified with an ExoSAP-IT. The PCR products were subjected to the Sanger method for DNA sequencing.

The microbial ecology of the frozen soil samples was evaluated via amplicon metagenomics. The analysis of the soil microbiome community consisted of the direct extraction of metagenomic DNA, and its amplification was performed by the PCR technique and sequencing^23^. The mixture of 10 pM of the amplifications was sequenced with MiSeq reagent 500V2 (Illumina) in the Illumina MiSeq apparatus, which generates sequences of 250 bases. DNA sequences were analysed using Quantitative Insights Into Microbial Ecology (QIME) versão 1.9.2 (Caporaso et al., 2010). The strings were grouped into Operational Taxonomic Units (OTUs) considering 97% identity with the 16S and 18S SILVA 132 database.

## Results and discussion

**Figure 2:**
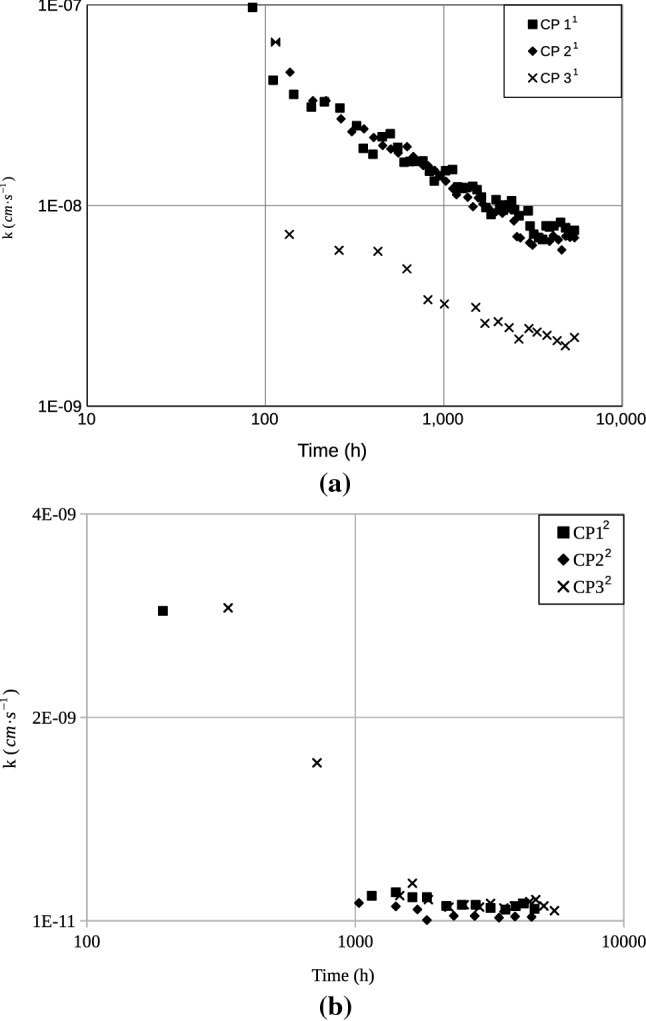
Permeability of soil samples. (**a**) First sampling campaign and (**b**) second sampling campaign.

**Figure 3:**
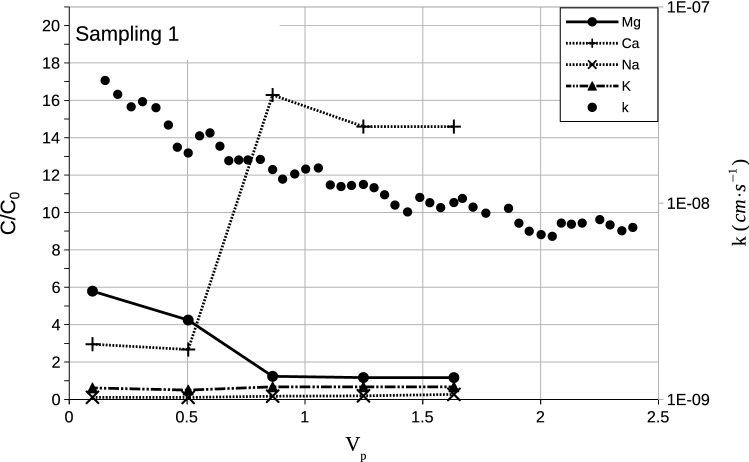
$\mathrm{C}/\mathrm{C}_0$ Breakthrough curves for cations and permeability.

Figure 2 presents hydraulic behaviour of the observed soil. As shown in the figure, all the samples presented a $\mathrm{k}$ decrease over time. Table 5 summarizes the results in terms of the initial $\mathrm{k}_{\mathrm{i}}$ average $\bar{\mathrm{k}}$, and final $\mathrm{k}_{\mathrm{f}}$ values of permeability and values of the ratio $\mathrm{k}_{{ \mathrm i}}/\mathrm{k}_{\mathrm{f}}$. Values of $\mathrm{k}_{\mathrm{i}}/\mathrm{k}_{\mathrm{f}}$ in the range from 3.9 to 28.3 were obtained. Figure 3 presents breakthrough curves for the main cations detected in PPW: Mg, Ca, Na, and K for sample $\mathrm{CP}2^1$. As observed, after $\mathrm{V}_{\mathrm{p}}=0.5$, the $\mathrm{Ca}$ concentrations present a sharp increase, while the $\mathrm{Mg}$ concentrations decrease and stabilize at values of $\mathrm{C}/\mathrm{C}_0\approx 1$ (soil no longer releases $\mathrm{Mg}$ after this point). Values of $\mathrm{C}/\mathrm{C}_0\approx 0$ are always obtained for $\mathrm{Na}$. The obtained behaviour for the cations breakthrough curves strongly corroborates the clay dispersion hypothesis^11^. Comparing the sampling groups, an initial sharp decrease in the values of $\mathrm{k}$ is observed for the samples from the second sampling campaign, which is followed by an approximately steady state phase that remains until the end of the test. The authors believe that this behaviour is probably linked to the higher sodium concentrations in the synthetic PPW.

**Table 5:** Initial, $\mathrm{k}_{\mathrm{i}}$, average, $\bar{\mathrm{k}}$ and final, $\mathrm{k}_{\mathrm{f}}$ soil permeability and $\mathrm{k}_{\mathrm{i}}/\mathrm{k}_{\mathrm{f}}$ ratios.

Column test	$\mathrm{k}_{\mathrm{i}}$ $(\mathrm{m} \cdot \mathrm{s}^{-1})$	$\mathrm{k}_{\mathrm{f}}$ $(\mathrm{m} \cdot \mathrm{s}^{-1})$	$\bar{\mathrm{k}}$ $(\mathrm{m} \cdot \mathrm{s}^{-1})$	$\mathrm{k}_{\mathrm{i}}/\mathrm{k}_{\mathrm{f}}$
CP $1^1$	9.7E−08	5.5E−09	9.4E−09	17.8
CP $2^1$	6.5E−08	5.4Ev09	8.6E−09	12.1
CP $3^1$	1.2E−08	1.5E−09	3.5E−09	8.4
CP $1^2$	3.1E−09	1.3E−10	4.5E−10	23.9
CP $2^2$	1.9E−10	4.8E−11	1.3E−10	3.9
CP $3^2$	3.1E−10	1.1E−10	4.6E−10	28.3

$^{1,2}$Samples from first and second sampling campaigns.

**Figure 4:**
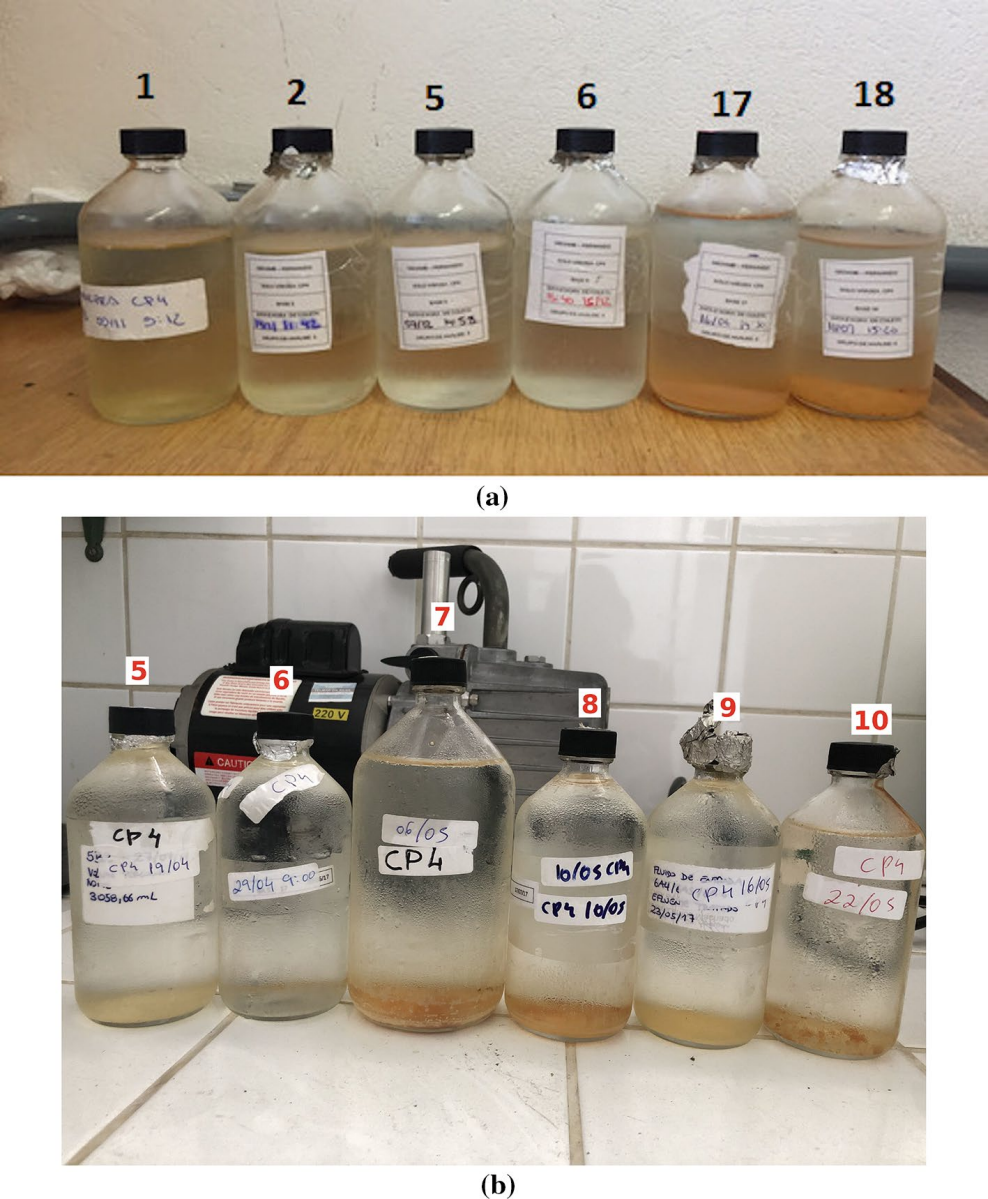
Photo of sampling jars containing percolated fluid. (**a**) First campaign and (**b**) second campaign.

Figure 4 illustrates the visual aspect of the collected effluent samples. Samples were enumerated according to the sampling order. Unlike the first effluent samples, samples collected at the end of the column tests presented a considerable amount of sediment at the bottom of the jars; As observed, however, clay dispersion seems to start earlier in samples from the second campaign, which is in accordance with the hydraulic behaviour presented in Fig. 2.

**Figure 5:**
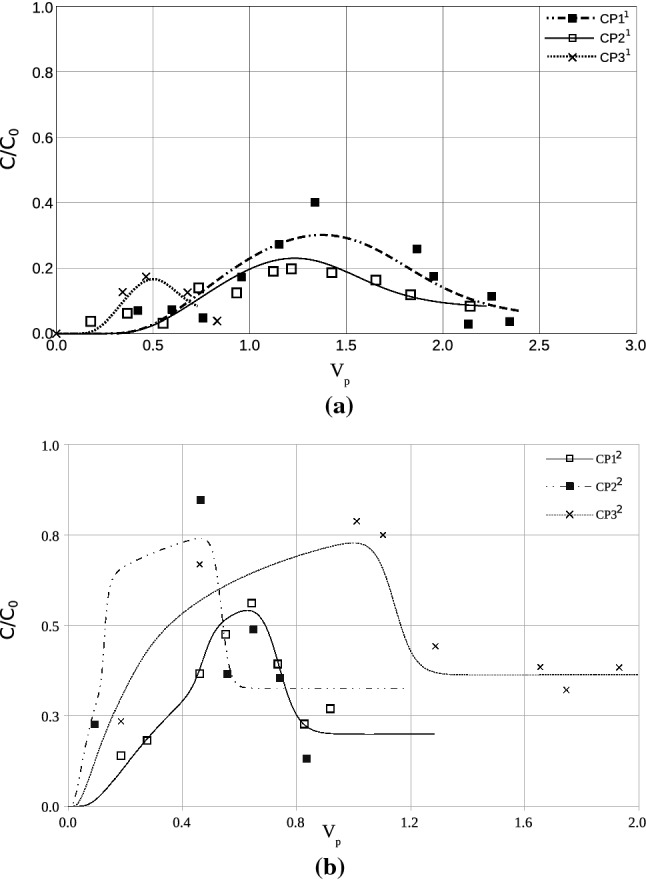
Experimental and fitted breakthrough curves. (**a**) First sampling campaign and (**b**) second sampling campaign.

Figure 5 shows the obtained experimental breakthrough curves whereas Table 6 summarizes the fitting parameters using Eq. (4). As presented in the figures, organic decay can be observed in all the performed tests. Despite the fact that the curves were obtained for the same soil, the fitting parameters presented noticeable differences between sampling campaigns.

**Table 6:** Obtained fitting parameters.

Column test	$\mathrm{P}_{\mathrm{L}}$	$\mathrm{R}_{\mathrm{d}}$	$\upalpha \,(\mathrm{m})$	$\uplambda _{\mathrm{max}}$ $(\mathrm{s}^{-1})$	$\upbeta$ $(\mathrm{s}^{-1})$	$\mathrm{t}_1\,(\mathrm{s})$	$\mathrm{R}^2$
CP $1^1$	0.35	2.0	1.10	1.5E−06	3.0E−07	4.0E06	0.82
CP $2^1$	0.78	1.7	0.50	9.0E−07	8.0E−07	1.0E06	0.88
CP $3^1$	0.62	1.5	0.60	5.0E−07	3.0E−07	5.0E06	0.92
CP $1^2$	0.45	1.6	0.88	5.5E−07	2.0E−06	1.1E07	0.93
CP $2^2$	0.20	1.3	2.00	5.9E−07	5.0E−06	1.1E07	0.74
CP $3^2$	0.36	1.4	1.08	3.5E−07	2.4E−06	1.4E07	0.96
Mean		1.6	1.00	7.3E−07	1.8E−06	7.7E06	
COV		0.14	0.48	0.52	0.91	0.60	

$^{1,2}$Samples from first and second sampling campaigns.

Regarding the organic decay process, higher values of $\mathrm{t}_1$ and $\upbeta$ were obtained for the samples from the second group. This delay in the development of the microbiological activities is probably linked to the lower permeability of the samples (refer to Table 5) and lower TPH concentrations in the synthetic PPW. In the case of aerobic degradation, the concentration of the substrate and transport of nutrients and oxygen in the soil are considered essential factors for the development of degradation activities. Lower permeabilities hinder the transport of these components to microbial cells and stimulate the development of anaerobic zones, which slow the degradation processes in the samples^24–26^. The maximum decay rate ($\uplambda _{\mathrm{max}}$), however, presented a smaller intergroup variability. This seems to be more dependent on the original nature and diversity in the communities of microorganisms in the soil, which were presumably similar.

**Figure 6:**
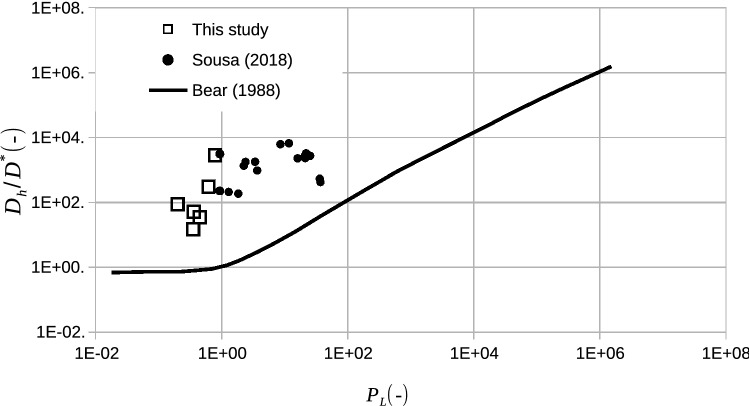
Hydrodynamic dispersion behaviour of column tests performed with PPW compared with values presented in the literature.

Figure 6 presents values of $D_{h} /D \times P_{L}$ obtained in the performed column tests and compares them with the curve presented by^13^, as a general trend for the ratio $\hbox{D}_{{\rm h}}/{\rm D}^*$ considering data from several different authors. $\hbox{D}_{{\rm h}}$ values obtained for PPW are approximately two orders of magnitude higher than values obtained for inorganic solutes. The obtained results endorse the previously mentioned aspects concerning the behaviour of the dissolved/dispersed constituents of the PPW.

Tables 7 and 8 present the most abundant genera obtained with the use of metagenomic techniques and compare the results with those obtained employing culture dependent methods with DNA sequencing. Lines starting with “*” indicate that the genus was identified both by metagenomic and culture dependent methods. The metagenomic analysis allowed the comparison between the soil microorganism before and after column tests. Bacteria were prevalent in relation to fungi and the most abundant genera are associated to hydrocarbon degrading activities. This was the case of the most abundant bacteria genus (*Delftia*), which is made up of aerobic bacteria which are capable of breaking down hydrocarbons of a greater molecular weight^27^. Other examples are *Mycoplana, Phenylobacterium, Fusobacterium, Rhodoplanes, Sphingomonas, Methylosinus, Novosphingobium* and *Staphylococcus*^28–31^. Considering the genera that could not be identified by the analysis on the QIIMI platform, the families Erythrobacteraceae, Comomonadaceae, and Xanthomonadaceae are associated with TPH decay^32–35^. The bacteria which can potentially breakdown hydrocarbons increased in number in the column tests compared to *in natura* soil. This suggests that they play an active role in the organic decay observed.

**Table 7:** Bacteria genera abundance.

Bacteria (Order: family: genus)	CP1$^2$ (%)	CP2$^2$ (%)	CP3$^2$ (%)	Mean (%)	“*In natura*” soil (%)
Burkholderiales: Comamonadaceae: *Delftia*	66.36	70.86	71.83	69.68	65.91
Acidobacteria:Not identified: Not identified: *Not identified*	3.13	0.41	4.34	2.63	0.00
Acidobacteria:Not identified: Not identified: *Not identified*	3.52	1.93	1.63	2.36	1.11
Burkholderiales:Comamonadaceae: *Not identified*	2.16	2.23	2.26	2.22	0.00
Burkholderiales: Comamonadaceae:*Not identified*	1.83	1.71	1.92	1.82	0.00
Saprospirales: Chitinophagaceae:*Sediminibacterium*	1.02	0.02	2.58	1.21	0.06
Xanthomonadales: Xanthomonadaceae: *Not identified*	2.01	0.81	0.16	0.99	0.43
Caulobacterales: Caulobacteraceae: *Mycoplana*	2.21	0.06	0.17	0.81	0.00
Acidobacteria:Not identified: Not identified: *Not identified*	1.63	0.22	0.47	0.77	0.49
Sphingomonadales:Erythrobacteraceae: *Not identified*	0.78	0.19	1.29	0.75	0.06
Not identified: Not identified: *Not identified*	1.22	0.08	0.29	0.53	0.00
Caulobacterales: Caulobacteraceae: *Phenylobacterium*	0.26	0.55	0.23	0.35	0.13
Fusobacteriales: Fusobacteriaceae: *Fusobacterium*	0.11	0.48	0.36	0.32	0.19
Rhizobiales: Hyphomicrobiaceae: *Rhodoplanes*	0.19	0.40	0.12	0.24	0.01
Rhizobiales: Methylocystaceae: *Methylosinus*	0.06	0.03	0.40	0.16	0.05
Sphingomonadales: Sphingomonadaceae: *Novosphingobium*	0.16	0.07	0.16	0.13	0.05
Bacillales: Staphylococcaceae: *Staphylococcus*	0.09	0.21	0.02	0.11	0.09
Sphingomonadales: Sphingomonadaceae: *Sphingomonas*	0.08	0.10	0.14	0.10	0.08
$^*$Bacillales: Bacillaceae: *Bacillus*	0.00	0.10	0.00	0.03	1.10
$^*$Bacillales: Bacillaceae : *Lysinibacillus*	0.00	0.10	0.00	0.03	0.10
$^*$Pseudomonadales: Pseudomonadaceae: *Pseudomonas*	0.01	0.00	0.01	0.01	0.02
$^*$Xanthomonadales: Xanthomonadaceae: *Stenotrophomonas*	0.01	0.07	0.02	0.04	0.02

**Table 8:** Fungi genera abundance.

Fungi (order: family: genus)	CP1$^2$ (%)	CP2$^2$ (%)	CP3$^2$ (%)	Mean (%)	“*In natura*” soil (%)
$^*$Malasseziales: Incertae Sedis: *Malassezia*	12.52	18.75	31.21	20.83	17.38
Agaricomycetes:Not identified: Not identified: *Not identified*	4.33	2.34	0.42	2.36	1.34
$^*$Eurotiales: Trichocomaceae: *Talaromyces*	1.66	1.79	3.47	2.30	7.61
Helotiales: Not identified: *Not identified*	5.71	0.00	0.00	1.90	0.00
Saccharomycetales: Incertae Sedis: *Candida*	0.00	0.00	2.31	0.77	0.00
Saccharomycetales: Not identified: Not *identified*	0.09	1.12	0.18	0.46	0.00
Pleosporales: Not identified: *Not identified*	0.74	1.95	0.00	0.90	0.00
Capnodiales: Not identified: *Not identified*	0.55	1.90	0.58	1.01	2.71
Agaricales: Not identified: *Not identified*	0.00	0.00	1.26	0.42	0.03
Onygenales: Not identified: *Not identified*	1.29	0.00	0.00	0.43	0.58
$^*$Hypocreales: Hypocreaceae: *Trichoderma*	0.50	0.00	1.00	0.50	0.00
$^*$Eurotiales:Trichocomaceae: *Aspergillus*	0.00	0.28	0.00	0.09	0.13

However, mainly in the case of bacteria, some of the most abundant genera were not detected using DNA sequencing after cultivation, isolation and oxidation tests. This is because only a small percentage of bacteria and fungi can be cultivated using traditional culture techniques^36^. Considering the culture tests performed on the fluid samples, a very low CFU number of bacteria and fungi was obtained (1,150 CFU/mL and 85 CFU/mL, respectively) compared to the tests performed in the soil samples. This indicates that the observed decay is probably due to the organisms present in the sediment.

The fungi genus *Malassezia* was the most abundant found in the metagenomic analysis, and it is one of the organisms that were able to degrade TPH in the performed oxidation tests. However, no indication in the consulted literature was obtained considering the possibility of organisms of the genus *Malassezia* degrading hydrocarbon compounds. This highlights the contribution of the present study. Considering the fungi of unidentified genus, the Eriotales, Helotiales and Agaricales orders and the family Aspergillus are reported as having hydrocarbon degrading potential. However, they did not perform well in the oxidation tests. Considering all the obtained results, four bacteria (*Bacillus* and *Lysinibacillus* genus) and two fungi species (*Malassezia* and *Talaromyces* genus) that were identified after the oxidation tests for hydrocarbon degradation have not previously been mentioned in the literature for having the ability to degrade TPH^37–39^.

## Conclusions

This paper presents results of column tests that were performed on compacted samples of a clayey sand soil permeated by PPW. The experimental results indicate the occurrence of clay dispersion in the samples with the associated decrease in soil permeability. Furthermore, all the tested samples presented the occurrence of organic decay. Breakthrough curves were modelled in an incremental way using a non constant decay rate to better simulate microorganisms growing process and consider the occurrence of varying velocity in experiments. A good adherence was obtained between experimental and modelled results. The TPH concentrations in the permeating fluid and/or soil permeability seem to influence the values of $\mathrm{t}_1$ (elapsed time for which the decay rate reaches half $\uplambda _{\mathrm{max}}$) and $\upbeta$ (rate of increase in the decay rate). The smaller variability of the maximum decay rate ($\uplambda _{\mathrm{max}}$) seems to reflect the similar original nature and diversity of the communities of microorganisms in the soil specimens^40^. The obtained $D_h$ values are substantially higher than the results presented in the technical literature concerning inorganic solutes but are compatible with previous results obtained using PPW as permeating fluid in different soils. The previously mentioned aspects are important (and most of them are favourable to environmental protection) when applying field remediation techniques or in the design of soil bio-barriers for PPW containment, since the VS sediment was characterized as a microbiota with potential to degrade hydrocarbons. The obtained results from the biological analysis illustrate how column tests can be performed to provide genetic material, which could be potentially applied in bio-remediation techniques that focus on native microorganisms. Furthermore, some identified species do not have a previous indication of the potential degradation of PPW compounds.
